# Silicate‐Phenolic Networks: Coordination‐Mediated Deposition of Bioinspired Tannic Acid Coatings

**DOI:** 10.1002/chem.201902358

**Published:** 2019-07-01

**Authors:** Florian Weber, Wei‐Chih Liao, Alejandro Barrantes, Mattias Edén, Hanna Tiainen

**Affiliations:** ^1^ Department of Biomaterials Institute of Clinical Dentistry University of Oslo, P.O. Box 1109 Blindern 0317 Oslo Norway; ^2^ Department of Materials and Environmental Chemistry Stockholm University 10691 Stockholm Sweden

**Keywords:** coordination polymers, polyphenols, surface chemistry, tannic acid/silicate complexation, thin films

## Abstract

Surface modification with polyphenolic molecules has been pursued in biomedical materials owing to their antioxidant, anti‐inflammatory, and antimicrobial characteristics. Recently, the use of silicic acid (Si_aq_) as a mediator for efficient surface deposition of tannic acid (TA) was reported, but the postulated Si‐TA polymeric networks were not characterized. Herein, we present unambiguous evidence for silicate‐TA networks that involve Si−O−C motifs by using solid‐state NMR spectroscopy, further supported by XPS and ToF‐SIMS. By using QCM‐D we demonstrate the advantages of Si_aq_, compared to using transition‐metal ions, to improve the coating efficiency under mildly acidic conditions. The presented homogenous coating buildup and validated applicability in inorganic buffers broadens the use of TA for surface modifications in technological and biomedical applications.

Polyphenolic molecules are well known for their antioxidant properties^1^ and thus have been utilized in biomedical applications as anti‐inflammatory,^2^ antimicrobial,^3^ and anticancer agents.^4^ Due to the substrate‐independent adhesive properties of catechol units, polyphenolic molecules have recently gained substantial attention toward creating novel bioinspired multifunctional material surfaces.^5^ Tannic acid (TA) is a naturally derived hydrolysable polyphenolic molecule consisting of five to ten galloyl units on a central glucose ring (Scheme 1),^6^ which account for its high antimicrobial and antioxidant capacity.^7^ Albeit the use of polyphenolic surface functionalization to overcome challenges in medicine and biotechnology has been proposed,^8^ to date, TA‐modified interfaces are only found in a few applications, such as controlled drug delivery^9^ and filtration membranes.^10^

**Scheme 1:**
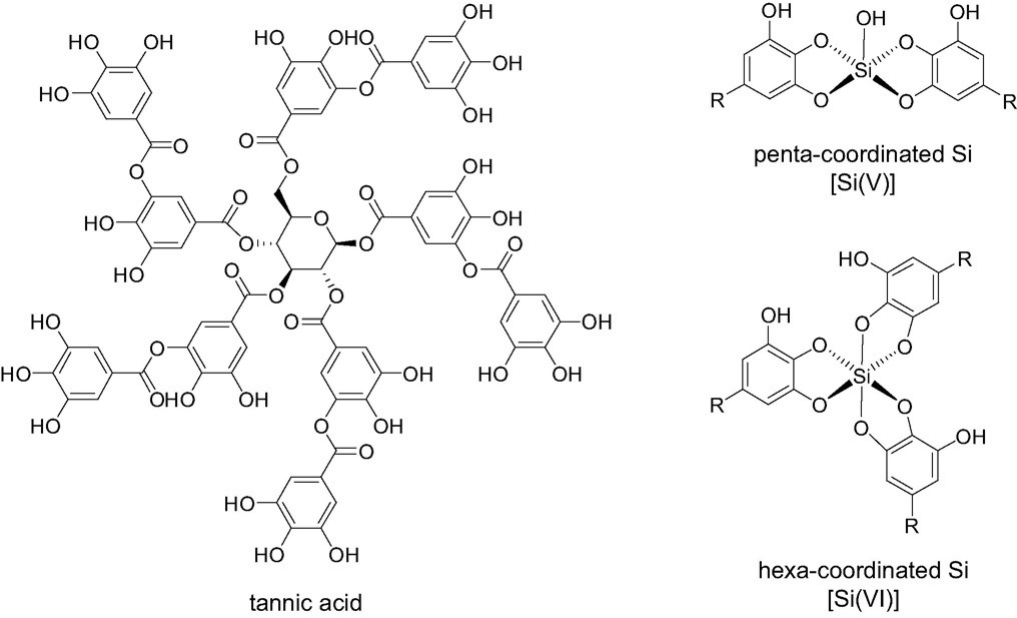
Schematic illustration of tannic acid (TA), penta‐coordinated Si [Si(V)] binding two TA ligands, and hexa‐coordinated Si [Si(VI)] binding three TA ligands.

Currently, two methods are applied to deposit TA onto surfaces: 1) self‐assembly of a metal phenolic network (MPN) and 2) induced oxidative polymerization. MPNs exploit the strong interaction between vicinal diol groups and transition‐metal ions.^11^ Usually, these systems are based on Fe^3+^ and are conducted in mildly alkaline conditions.^12^ Although MPNs have become the predominant method to create TA coatings, their drawback lies in the deposition of only one molecular layer per deposition cycle and the formation of complexation byproducts in solution.^13^ Efforts have, therefore, been made to induce a continuous coating formation by slow conversion of Fe^2+^ to Fe^3+^, yet with limited efficiency.^14^ Induced oxidative polymerization is based on the spontaneous auto‐oxidative polymerization of polyphenols in an alkaline environment3c, 15 or triggered by UV‐light.^16^ The stability of TA in alkaline conditions is, however, limited, and the auto‐oxidation by dissolved oxygen leads to uncontrolled degradation of TA and precipitation of polymeric byproducts.^17^

Recently, we reported an alternative deposition method using silicic acid (Si_aq_), which enables a continuous TA coating formation on titanium surfaces.^18^ However, the structural role of Si_aq_ in the coating formation remained unknown. Herein, we provide direct evidence for the formation of silicate–TA networks by magic‐angle spinning (MAS) NMR spectroscopy. These results are supported by X‐ray photoelectron spectroscopy (XPS) and time‐of‐flight secondary ion mass spectroscopy (ToF‐SIMS). Based on the formation of silicate–TA networks, we present a novel deposition method in mildly acidic conditions for improved TA stability in solution compared to using transition‐metal ions or oxidative conditions. By introducing a continuous‐flow process and demonstrating the TA coating formation in inorganic buffers, we extend the applicability of TA coatings for technological and biomedical purposes, such as designing modified implant surfaces with reduced infection risk.^2^

We employed ^1^H→^29^Si cross‐polarization (CP) MAS NMR to investigate the coordination state of Si in two samples prepared with 99.7 % ^29^Si‐enriched silicate (^29^Si_aq_; see the Supporting Information): TA‐coated TiO_2_ particles (TA_coating_) prepared in an 80 μm ^29^Si_aq_ solution, and TA precipitated with 1000 μm ^29^Si_aq_ (TA_prec_). The ^29^Si CPMAS NMR spectra shown in Figure 1 reveal nearly identical ^29^Si responses, which justifies using the Si‐richer TA_prec_ specimen for the remaining NMR experimentation. From previously reported ^29^Si chemical shifts involving Si−O−C bonds,^19^ the two peaks at −99 and −139 ppm of Figure 1 are assigned to ^29^Si coordinating five [Si(V)] and six [Si(VI)] phenolic O atoms, respectively, thereby complexing two and three galloyl motifs of TA (Scheme 1). These assignments are corroborated by the more rapid NMR‐signal buildup observed from the ^29^Si(V) sites (Figure S2, Supporting Information), the direct Si−OH bond of which implies a shorter ^29^Si−^1^H distance than their ^29^Si(VI) counterparts, which solely feature Si−O−R motifs (Scheme 1). Further evidence for silicate‐TA complexation is provided by the ^13^C{^29^Si} rotational‐echo double‐resonance (REDOR)^20^ NMR results in Figure S3, of the Supporting Information. A significant ^13^C NMR‐signal attenuation is only observed from the aromatic ^13^C moieties, meaning that they feature shorter internuclear distances to ^29^Si than all other ^13^C sites. This result accords with Si binding to the vicinal phenolic O positions of the TA molecule, as proposed in Scheme 1.

**Figure 1:**
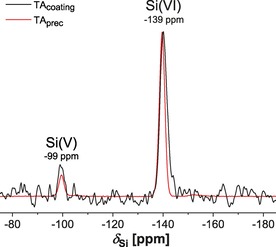
Solid‐state ^29^Si CPMAS NMR spectra recorded at 9.4 T and 7.00 kHz MAS from TA‐coated TiO_2_ particles in the presence of 80 μm ^29^Si_aq_ solution (TA_coating_), and from TA precipitated from a 1 mm ^29^Si_aq_ solution (TA_prec_). The NMR peaks at *δ*_Si_=−99 and *δ*_Si_=−139 ppm are assigned to ^29^Si species coordinating five [Si(V)] and six [Si(VI)] phenolic O atoms of the TA ligands, respectively (Scheme 1).

Both ^29^Si NMR (Figures 1 and Figure S1, Supporting Information) and XPS (Figure S6) evidenced negligible SiO_2_ contents in the TA coating. Hence, we conclude from Figure 1 and the integrated ^29^Si NMR peak intensities of the quantitative ^29^Si NMR spectrum of Figure S1 (Supporting Information) that ≈90 % of all Si is hexa‐coordinated by phenolic O atoms in the TA_prec_ and TA_coating_ samples. Moreover, XPS and ToF‐SIMS mappings verified that Si is distributed evenly across the polymeric TA network (Figure S8, Supporting Information).

Figure S4 shows ^13^C CPMAS NMR spectra recorded from pristine TA (TA_ref_), TA_prec_, as well as oxidized TA (TA_ox_), which was formed in a Si‐free buffer solution at pH=7.8. The NMR responses from TA_prec_ and TA_ref_ are similar, in which the latter accords with a previous report.^21^ The main distinction is the emergence of a resonance at ≈150 ppm in the ^13^C NMR spectrum of TA_prec_ that is attributed to ^13^C−O−Si fragments based on the ^13^C shift^22^ and our ^13^C{^29^Si REDOR NMR results (Figure S3, Supporting Information). The ^13^C NMR peaks between 50 ppm and 80 ppm stem from the central glucose ring,^21^ indicating an overall intact structure of TA upon its complexation with Si. In contrast, TA_ox_ revealed a distinctly different ^13^C NMR spectrum (Figure S4, Supporting Information). The absence of resonances below 80 ppm suggests either an oxidation of the glucose ring or a cleavage of gallic acid ester bonds to form gallic acid residues. Either scenario is consistent with the ^1^H NMR signal at 15.5 ppm observed from the TA_ox_ sample (Figure S5, Supporting Information), which is attributed to hydrogen‐bonded acidic protons.

Identification of the formation of Si−O−C motifs, as well as successfully depositing TA under a N_2_ atmosphere (Figure S9, Supporting Information), demonstrated that the complexation between Si_aq_ and TA constitutes the deposition of the polyphenolic network. Consequently, it shows that, unlike for other polyphenolic molecules, oxidative polymerization by dissolved O_2_ is not required.^23^ Given that oxidation of TA is associated with the formation of polymeric byproducts, both coating homogeneity and deposition efficiency benefit from restricting the oxidative polymerization. Therefore, we adjusted the solution pH and monitored the coating process in real‐time (Figure S10, Supporting Information) using a quartz crystal microbalance (QCM‐D). Figure 2 shows the thickness of TA coatings formed on titanium sensors under different pH conditions. For pH>8.2, the rapid polymerization of TA impeded the deposition process. At pH=7.8, the coating thickness was in accordance with previously reported values15a and the change from Bicine to HEPES did not result in a major deviation. By reducing the pH of HEPES, an increase in the coating thickness was observed as a result of reduced oxidation (Figure S11, Supporting Information). In contrast, a limited solubility of TA in BisTris manifested in a significantly lower coating thickness at pH=7.0 compared to the equivalent coating formation in HEPES.

**Figure 2:**
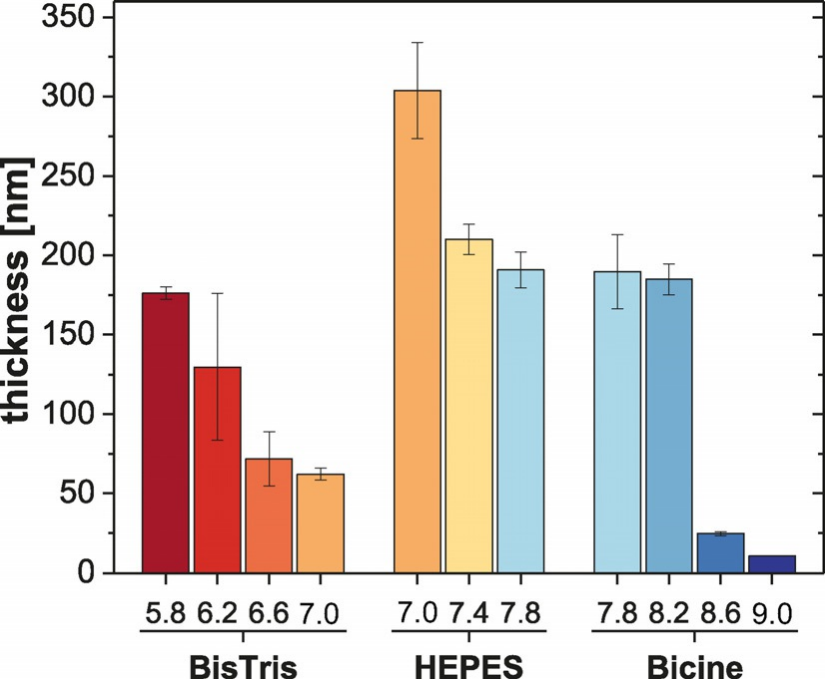
Averaged (*n*_rep_=3) film thickness of TA coatings measured by QCM‐D. The obtained values show the Voigt modeled thickness after 24 h adsorption time at different pH. Auto‐oxidative polymerization impeded the deposition at pH>8.2 and data represents the thickness before polymeric byproduct formation.

Since HEPES buffer covers both oxidizing and nonoxidizing pH conditions (Figure S11, Supporting Information), we characterized the structural properties of TA coatings at pH values of 6.8 and 7.8. The progression of the frequency and dissipation shifts at pH=7.8 (Figure 3) attested that the adsorption of TA leveled out after 8 h. This effect is more perceivable in dissipation versus frequency (Δ*D*/Δ*F)* plots in which the deposition process of TA showed three distinct phases. An initial horizontal decrease of Δ*F* (Figure 3 B, I) indicates a rigid layer. Subsequently a transition phase (II) resulted in increased viscoelastic properties until the third regime is reached, in which a vertical progression (III) denotes the increasing dissipative properties. Using nanoplasmonic spectroscopy (NPS), we further studied the initial coating formation in detail (Figure S13, Supporting Information). The comparison of the optical mass to the acoustic mass confirmed a low hydration of the TA layer during the first 30 min, followed by a gradual increase to ≈30 % after 1 h (Figure S12, Supporting Information). Similarly, plotting Δ*λ* against Δ*F* and Δ*D* results in its structure–characteristic curve shape. Extending the analysis of NPS dry mass, we determined the layer thickness by means of ellipsometry and AFM. After 24 h, an in situ thickness of 191±11 nm was obtained, which corresponded to 158±3 nm (AFM: 132±8 nm) in a dry state. From the lower dry state thickness, we conclude that the hydrated layer collapses and forms a rigid layer upon drying.

**Figure 3:**
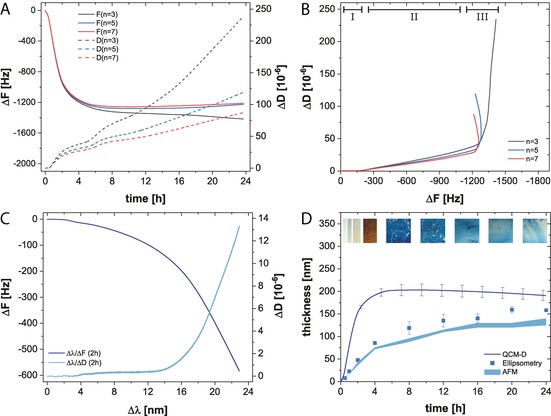
Deposition of TA coatings from HEPES at pH=7.8. (A) Averaged (*n*_rep_=3) and normalized frequency (Δ*F*) and dissipation shifts (Δ*D*) of the 3rd, 5th, and 7th QCM‐D overtone (*n*) as a function of time and the correlated frequency versus dissipation plot (B). (C) Correlation between optical and acoustic mass. (D) In situ thickness compared to dry thickness of TA coatings (Inset: coated Si wafer).

In buffered solution at pH=6.8 (Figure 4), the initial adsorption kinetics was considerably slower compared to pH=7.8. This may emanate from base catalytical processes, or from O−H dissociation of either silicic acid (p*K*_A_=9.8) or TA (p*K*_A_=9.9).19c However, compared to the deposition kinetics at pH=7.8, no leveling out after 8 h was observed. In Δ*D*/Δ*F* plots, a clear difference is noticeable, manifested in the absence of the third regime. It is likely that less TA reacts in oxidative polymerization processes at pH=6.8 and thus more TA is available for the coating deposition, which led to a more homogenous layer thickness of 266±2 nm after 24 hours in situ. Our combined assessment of the optical and acoustic mass revealed that the layer hydration was equivalent to the layer obtained at pH=7.8 (Figure S12, Supporting Information). The time‐decoupled plot of Δ*λ* against Δ*F* and Δ*D* resulted in the same characteristic curve shape and ascertained negligible structural differences between TA coatings obtained in either pH condition. Ellipsometry determined a thickness of 231±9 nm (AFM: 180±10 nm) after 16 h and confirmed the higher efficiency at pH=6.8.

**Figure 4:**
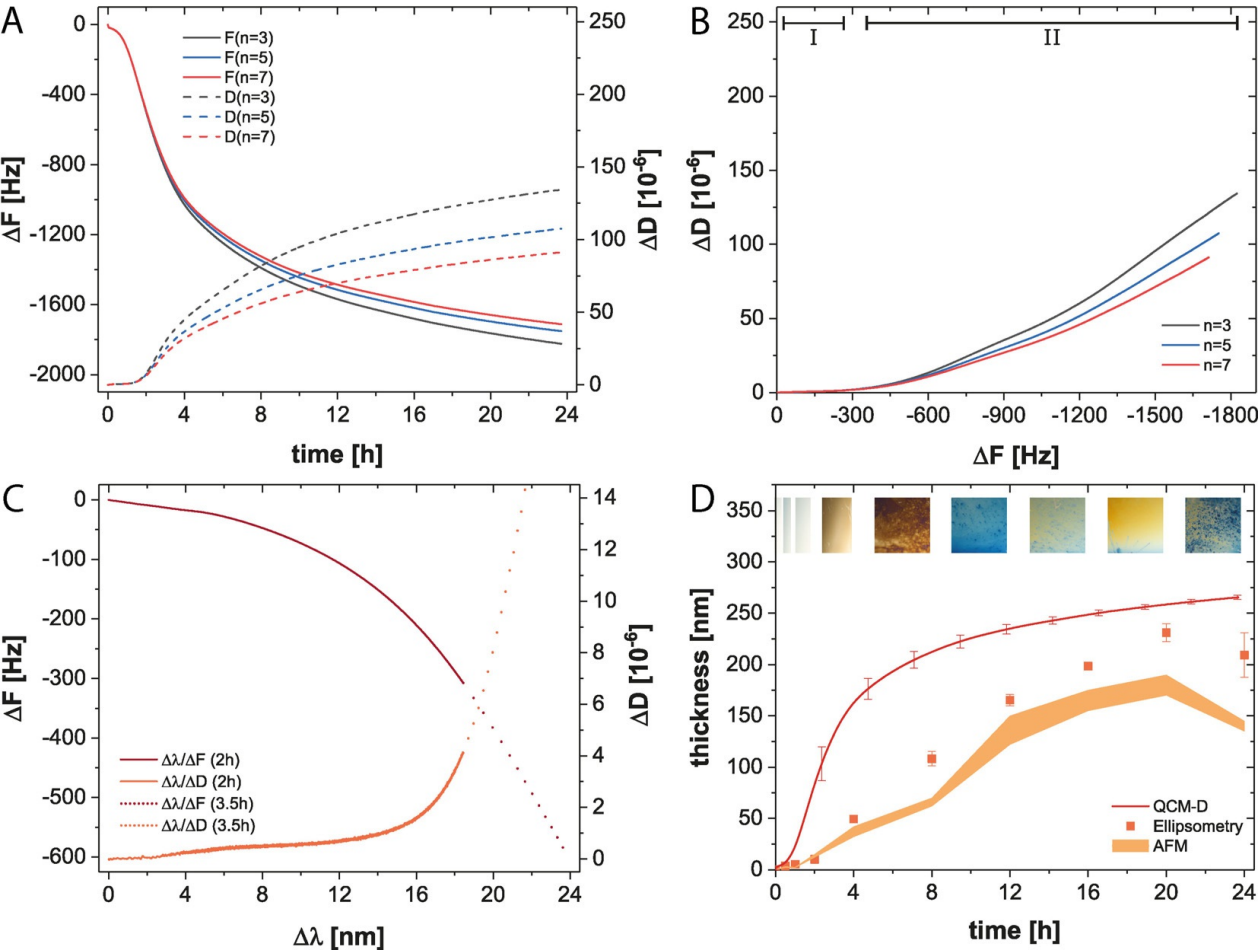
Deposition of TA coatings from HEPES at pH=6.8. (A) Averaged (*n*_rep_=3) and normalized frequency (Δ*F*) and dissipation shifts (Δ*D*) of the 3rd, 5th, and 7th QCM‐D overtone (*n*) as a function of time and the correlated frequency versus dissipation plot (B). (C) Correlation between optical and acoustic mass. (D) In situ thickness compared to dry thickness of TA coatings (Inset: coated Si wafer).

With an improved deposition efficiency, TA nanocoatings can be pushed towards microscale dimension and the deposition of higher amounts of TA is possible. Thereby the larger reservoir of TA may enable improved antibacterial and anti‐inflammatory effects of TA coatings.3b, 5c In order to break the boundaries of nanoscale polyphenolic coatings, the increased coating kinetics in alkaline conditions is a crucial requirement. Simultaneously, the formation of polymeric TA byproducts in solution must be avoided.

With Si as the coordinating species, the coating process can be changed from a batch reaction to a continuous‐flow process (Figure S14, Supporting Information). By separating TA from Si_aq_, TA can be kept stable at pH=6.8 and fed with Si_aq_ at pH=8.8, yielding a quadrupled TA‐deposition compared to the batch process shown in Figure 3.

Moreover, we investigated the deposition process in alternative buffer systems for applications in which organic buffer molecules interfere with other chemical reactions. We demonstrated that TA coatings can be deposited in both citrate/phosphate and pure phosphate buffered solutions (Figure S15, Supporting Information). Tuning of the reaction speed can finally be performed by adjusting the pH and the ionic strength of the buffer.15a, 24

In conclusion, we have presented direct experimental evidence for a complexation between silicate and TA that contributes to the deposition of TA coatings. The overall structurally intact TA molecules are expected to retain the antioxidant properties of the coating. By optimizing the solution pH, a prolonged and more homogenous deposition process was achieved. By demonstrating a continuous‐flow process yielding high deposition rates, we establish a method that is commensurate with industrial demands, while giving a low rate of byproduct formation. For applications interfering with organic buffers, we have expanded the deposition of TA to inorganic buffers, which may open the utilization of TA coatings on implantable biomedical devices to prevent biofilm‐associated infections and to improve the host tissue integration.

## Experimental Section

Details of materials and methods, along with supplementary experimental data, can be found in the Supporting Information.

## Conflict of interest

The authors declare no conflict of interest.

## Supporting information

As a service to our authors and readers, this journal provides supporting information supplied by the authors. Such materials are peer reviewed and may be re‐organized for online delivery, but are not copy‐edited or typeset. Technical support issues arising from supporting information (other than missing files) should be addressed to the authors.

Supplementary
