# Supplementary material for: Silicate‐Phenolic Networks: Coordination‐Mediated Deposition of Bioinspired Tannic Acid Coatings
Source: Chemistry. 2019 Jul 1;25(42):9870–4. doi: 10.1002/chem.201902358 (PMC6772174; doi:10.1002/chem.201902358)
Supplement: Supplementary file 1 — Supplementary [file CHEM-25-9870-s001.pdf]

CHEMISTRY
A European Journal

Supporting Information

Silicate-Phenolic Networks: Coordination-Mediated Deposition of Bioinspired Tannic Acid Coatings

Florian Weber,[a] Wei-Chih Liao,[b] Alejandro Barrantes,[a] Mattias Edén,[b] and Hanna Tiainen*[a]

chem_201902358_sm_miscellaneous_information.pdf

Silicate-Phenolic Networks: Coordination Mediated Deposition of Bioinspired Tannic Acid Coatings

Table of Contents

Experimental section....2
- Materials....2
- Coating Preparation....2
- Solid-State NMR....3
- XPS....4
- EDS....5
- ToF-SIMS....5
- QCM-D....5
- NPS....5
- Ellipsometry....6
- AFM....6
- UV-vis....6

Supporting information for solid-state NMR measurements....7
Supporting information for surface analysis....10
- XPS....10
- Si distribution determined by XPS, ToF-SIMS, and EDS....11

Supporting information for deposition of tannic acid coatings....12
- QCM-D raw data....12
- Spectrophotometric determination of TA oxidation....14
- NPS data....14
- CSTR type deposition of TA....15
- TA deposition from inorganic buffers....16

CSTR setup....16
References....17

[a] F. Weber, Dr. A. Barrantes, Prof. H. Tiainen,
Department of Biomaterials, Institute of Clinical Dentistry,
University of Oslo,
P.O. Box 1109 Blindern, 0317 Oslo, Norway,
E-mail: hannati@odont.uio.no

[b] Dr. W.-C. Liao, Prof. M. Edén,
Department of Materials and Environmental Chemistry,
Stockholm University,
SE-106 91 Stockholm, Sweden

Experimental section

Materials

Tannic acid (ACS grade, MW = 1701.2, LOT#MKBN9606V) and sodium metasilicate pentahydrate (≥ 95%) were purchased from Sigma Aldrich. Unless otherwise stated, all materials were ACS grade and purchased from VWR.

Coating Preparation

Organic buffers were prepared by dissolving Bicine (≥ 99%, Sigma Aldrich), HEPES (BioPerformance, ≥ 99.5%, Sigma Aldrich), or BisTris (BioPerformance, ≥ 98%, Sigma Aldrich) at a concentration of 100 mM together with 600 mM NaCl in MilliQ water (18 MΩ cm). The pH was subsequently adjusted with 10 M NaOH or 5 M HCl to the needed level. NaOH stock solutions and prepared buffer solutions were stored in plastic bottles to avoid dissolving any silica ions from glassware.

Citrate buffer was prepared from 100 mM citric acid monohydrate (≥ 98%, Sigma Aldrich) and 200 mM disodium hydrogen phosphate (Na_2HPO_4, 98 – 102%; Sigma Aldrich) at a volume ratio of 17.6/82.34 for a final pH of 7.0. The buffer was supplemented with 600 mM NaCl and deviations from pH = 7.0 were corrected with 10 M NaOH or 5M HCl.

Phosphate buffer was prepared from 200 mM sodium dihydrogen phosphate (NaH_2PO_4, 98 – 102%; Sigma Aldrich) and 200 mM disodium hydrogen phosphate (Na_2HPO_4, 98 – 102%; Sigma Aldrich) at a volume ratio of 19.5/30.5. The solution was diluted with an equal amount of MilliQ water and supplemented with 600 mM NaCl. Deviations from pH = 7.0 were corrected with 10 M NaOH or 5 M HCl.

Tannic acid was dissolved in buffer solutions at a concentration of 1 mg/ml and stirred until fully dissolved. The concentration of 80 μM ortho-silicic acid (Si_{aq}) was adjusted from a 0.1 M stock solution of sodium metasilicate pentahydrate dissolved in MilliQ water.

Coatings prepared under continous flow conditions (CSTR setup) were obtained by seperating TA from Si_{aq} (Figure S16). TA at a concentration of 2 mg/ml was kept at acidic pH = 6.8 to prevent oxidation, while Si_{aq} at a concentration of 160 μM was adjusted to pH = 8.8. Both solution were mixed in a subsequent beaker (V_R = 6 ml) at a flow rate of 0.1 ml/min resulting in an average residence time of τ = 30 min. The solution pH in the CSTR was monitored and deviations from pH = 7.8 were adjusted by the pH level of the Si_{aq} feed.

As coating substrates, either polished titanium coins (grade IV) or silicon wafers (n-type, (100), Sigma Aldrich) were immersed in solution under gentle stirring. Prior to the coating process, Si wafers and Ti coins were treated for 15 min in an UV-Ozone chamber (Novascan PSD-UV4). Subsequently, the substrates were cleaned in 5/1/1 mixture of H_2O, 30% ammonia, 30% H_2O_2 to remove organic contaminants. Si wafers were finally treated with 10% HF for 10 min to create hydrophobic surfaces.

Chemical analytical experiments (XPS, EDS, ToF-SIMS) were conducted on Ti coins, which were coated with TA for 24 h in Bicine buffer at pH = 7.8 in presence of 80 μM Si_{aq}. AFM and ellipsometric measurements of TA coating thicknesses were evaluated on Si wafers. Note the different preparation procedures for the samples employed for the solid-state NMR experiments, as described below.

Solid-State NMR

Given the low Si contents in the samples (Table S3), all ^{29}Si NMR experimentation was conductedd on ^{29}Si-enriched samples, prepared using $^{29}SiO_2$ (99.7% enrichment; BuyIsotope, Neonest AB, Sweden) dissolved 1.00 ml 10 M NaOH and added to HEPES buffer. The amounts of added $^{29}SiO_2$ yielded nominal Si concentrations of 80 μM and 1000 μM in the preparation of the "*TA precipitate"* (TA_{prec}) and the "*TA coating"* ($TA_{coating}$) specimens, respectivly. The pH of the solution was then adjusted to 7.8 using 10 M NaOH.

TA_{prec} was collected by filtering the reaction solution through a 0.2 μm nitrocellulose membrane after 24 h. The $TA_{coating}$ specimen was prepared by coating TiO_2 to mimic the native oxide layer of titanium surfaces described in the other experimental sections. The particles (sieved fraction with particle diameter between 100–180 μm) were suspended at a concentration of 1.5 mg/ml in TA solution for 1 h to avoid additional TA particle formation, followed by filtering through a nitrocellulose membrane (0.2 μm). The coated TiO_2 particles were subsequently washed with HEPES buffer solution. "*TA oxidation products"* (TA_{ox}), which formed at pH = 7.8 during 24 h in reaction solutions that did not contain any silicon, were collected by filtration (0.2 μm membrane). For comparison, the *pristine TA* precursor powder (TA_{ref}) was analyzed as received.

All ^{1}H, ^{13}C, and ^{29}Si MAS NMR experimentation was performed with Bruker Avance-III spectrometers at static magnetic fields (B_0) of 9.4 T or 14.1 T, which correspond to the respective ^{1}H/^{13}C/^{29}Si Larmor frequencies of 400.1/100.6/79.5 MHz and 600.1/150.9/119.2 MHz. Neat tetramethylsilane (TMS) was used to calibrate each ^{1}H, ^{13}C, and ^{29}Si chemical shift at 0 ppm. Fine powders of the TA_{prec}, $TA_{coating}$, TA_{ox}, and TA_{ref} samples were packed in 4 mm (outer diameter) zirconia rotors and ceiled with Kel-F caps. Throughout all Figures, all NMR spectra are for visualization purposes zoomed so as to comprise all relevant NMR signals, i.e., to emphasize the significant information.

Directly excited ("single-pulse") ^{1}H and ^{29}Si NMR spectra were recorded at 9.4 T and the MAS rate ν_r = 14.00 kHz, using 90° radiofrequency (rf) pulses operating at rf nutation frequencies of 54 kHz and 100 kHz for ^{29}Si and ^{1}H, respectively, with corresponding relaxation delays of 300 s and 5 s. These relaxation delays were selected from separate T_1 relaxation experiments to ensure quantitative NMR spectra. The total numbers of accumulated signal transients were 256 for ^{29}Si and 8 for ^{1}H. No ^{1}H decoupling was applied during the ^{29}Si NMR signal acquisitions.

^{1}H→^{13}C and ^{1}H→^{29}Si cross polarization (CP) NMR experiments performed at B_0 = 9.4 T employed MAS rates of 14.00 kHz for ^{13}C and 7.00 kHz for ^{29}Si. CP was established at the modified Hartmann-Hahn conditions

$$\nu_1^{\mathrm{H}} - \nu_1^{X} = n\,\nu_r, \qquad X = \{^{13}\mathrm{C}, ^{29}\mathrm{Si}\},$$

employing proton nutation frequencies ν_1^{H} of 43 kHz (*n* = −1) and 64 kHz (*n* = 1) for the ^{29}Si and ^{13}C NMR experiments, respectively. For both ^{29}Si and ^{13}C, the nutation frequency was ramped linearly[1] by ±5% around ν_1^{X} = 50 kHz. Contact-time periods of 4.0 ms and 2.0 ms were employed for all ^{29}Si and ^{13}C experiments, respectively (except for those shown in Figure S2). Heteronuclear ^{1}H decoupling during the NMR-signal acquisitions utilized the SPINAL-64 scheme[2] operating at ν_1^{H} = 83 kHz (5.8 μs ^{1}H pulses). Relaxation delays of 1.0 s were used for all ^{13}C CPMAS NMR experiments, whereas those involving ^{29}Si used 1.5 s and 3.0 s for the TA_{prec} and $TA_{coating}$ samples, respectively. Depending on the detected nucleus and sample,

6000–65000 accumulated transients were required to obtain NMR spectra of moderate to high quality (see Table S1).

The $^{13}C\{^{29}Si\}$ rotational-echo double-resonance (REDOR)[3] NMR experiments on the TA_{prec} specimen were performed at B_0 = 14.1 T and ν_r = 10.00 kHz, starting from ^{13}C magnetization generated by ramped $^1H \rightarrow ^{13}C$ CP, employing ν_1^{H} = 42 kHz, ν_1^{C} = 32 kHz, and a contact time period of 2.0 ms. The REDOR protocol involves recording two separate NMR data sets for a given dipolar recoupling interval τ_{rec}:[3] (*i*) a “reference” NMR spectrum [$S_{ref}(\tau_{rec})$], which involves a Hahn-echo with a 180° rf-pulse (14.0 μs in our experiments) applied to the observed nuclei (^{13}C) to refocus chemical shifts; (*ii*) a “dipolar-dephased” spectrum [$S(\tau_{rec})$], for which additionally a rotor-synchronized train of 180° rf pulses (13.6 μs; ν_1^{Si} = 37 kHz) is applied to ^{29}Si in order to reintroduce the MAS-averaged ^{13}C–^{29}Si dipolar interactions.[3] Their presence results in an attenuation (“dephasing”) of the ^{13}C NMR signals from all ^{13}C sites in close spatial proximity to ^{29}Si. Our experiments used τ_{rec} = 4.0 ms, with the rf phases of the 180° recoupling pulses cycled according to the XY8 scheme.[4] In all REDOR experiments, heteronuclear proton decoupling (ν_1^{H} = 67 kHz) were accomplished using continuous-wave (CW) and SPINAL-64[2] schemes during the dipolar-recoupling and NMR-signal acquisition periods, respectively. The relaxation delays were 3.0 s, and 12800 signal transients were collected for each part of the REDOR protocol.

Table S1: Number of accumulated transients used for the ^{13}C and ^{29}Si CPMAS NMR experiments.

Figure label	Sample	Number of transients
Figure 1	$TA_{coating}$	68408
	TA_{prec}	6144
Figure S1	TA_{prec}	6144
Figure S2	TA_{prec}	256 (512 for the contact time of 0.3 ms)
Figure S4	TA_{ref}	47752
	TA_{prec}	57344
	TA_{ox}	36864

XPS

Analysis of the silicon content and distribution was conducted on a Kratos XPS instrument (Kratos Analytical Ltd). Sprectra were obtained with a monochromated Al K_{α} radiation (15 kV, 10 mA) under charge compensation (2 A, 3.8 V). Survey spectra were measured with a pass energy of 160 eV, a step size of 1 eV, and a 200 ms dwell time. High-resolution spectra were obtained with a pass energy of 20 eV, a step size of 0.1 V, and a dwell time of 1000 – 2000 ms. Maps were acquired in parallel XPS imaging mode at a spatial resolution of 3 μm. The acquisition time was set to 300 s with a pass energy of 160 eV.

The data was processed in CasaXPS (Casa Software Ltd) by shifting the C_{1s} peak with the lowest binding energy to 285 eV. Peak fitting and deconvolution were performed with a Shirley background and a symmetric Gaussian/Lorentian line shape.

EDS

Coated Ti coins were analyzed regarding the atomic composition and surface morphology using an FEI Quanta 450 scanning microscope (Thermo Fisher Scientific) coupled to an X-Max^N Oxford 50 mm^2 analyzer (Oxford Instruments).

ToF-SIMS

ToF-SIMS analysis was performed using a PHI TRIFT V nanoTOF instrument (Ulvac-Phi Inc.) equipped with a 30 keV LIMG source. The primary ion beam (Ga^+) was set to the unbunched mode to be optimized for high lateral resolution (< 0.4 μm). The ^{28}Si isotope was detected in positive mode with an analyzed area of 100 μm × 100 μm and 30 μm × 30 μm in size. No charge compensation was required.

QCM-D

A QSense E4 (Biolin Scientific) quartz crystal microbalance (QCM-D) was used to monitor the real-time formation of TA nanocoatings. Ti sensors (QSX 310, Biolin Scientific) were used and cleaned according to the manufacturer's protocol before and after each experiment. The procedure includes sonication in 2% SDS, washing with MilliQ water and EtOH, and final UV-ozone treatment. The QCM-D chambers were cleaned with 2% SDS for 10 min and extensively flushed with water (> 15 min) prior to the experiment.

Before adsorption of tannic acid, sensors were equilibrated in buffer and a baseline was recorded. TA was flown through the cell at 0.1 ml/min at 21°C under gentle stirring of the solution (100 rpm). After the adsorption, the sensors and chambers were flushed with MilliQ water and 0.1 M HCl for 5 min to remove tannic acid. Subsequently, the chamber cleaning protocol was conducted as described before. All experiments were performed in triplicates ($n_{rep} = 3$).

The change in oscillation frequency (ΔF) and dissipation (ΔD) was continously monitored for the fundamental frequency and five overtones. For clarity, only the first three overtones are plotted. Calculations of layer thicknesses were performed with QTools Software (BiolinScientific, Version 3.1.33) using the extended viscoelastic model. Fits were obtained based on the 3rd ($n = 3$), 5th ($n = 5$), and 7th ($n = 7$) harmonic overtone and a power based shear dependence. The TA layer density was assumed to be 1046 kg/m^3,[5] accompanied by a measured fluid density of 1027 kg/m^3.

NPS

Nanoplasmonic spectroscopy (NPS) is a technique based on the concept of measuring the localized surface plasmon resonance (LSPR) and was conducted on an Acoulyte (Insplorion AB) instrument. The instrument allows simultanous recording of the optical mass and acoustic mass using the QCM-D equipment. TiO_2 coated sensors, provided by the manufacturer, were immersed in 2% SDS for 10 min, washed with MilliQ water, and UV-ozone treated prior to the experiment.

The coating procedure is performed as described in the QCM-D section. In short, sensors were equilibrated in buffer before TA was flown through the cell at 0.1 ml/min at 21°C. All experiments were conducted as duplicates (n_{rep} = 2).

The calculation of the optical mass was obtained with respect to the exponential decay of the plasmon signal (Equation S1). The adsorbed areal mass (Γ) depends on the layer thickness (d_S) obtained from QCM-D, the surrounding refractive index (n_{buffer}), and the refractive index (n_S) and the refractive index increment (dn/dc) of the layer. The difference in refractive index (Δn) is correlated with the change of the plasmon peak ($\Delta\lambda$), the sensitivity factor of the TiO_2 sensor (S_0 = 140 nm/RIU), and the characteristic decay length of the surface plasmon (L_Z = 30 nm).[6] The refractive index increment was determined to be 0.173 ± 0.01 ml/g.

$$\Gamma = d_s \frac{\Delta n}{dn/dc} = d_s \frac{\Delta\lambda}{S_0\left(1 - e^{\frac{-2d_s}{L_z}}\right) \cdot dn/dc} \quad \text{(S1)}$$

Ellipsometry

Coated Si wafers were analyzed using a Rudolf Auto EL III null ellipsometer (Rudolf Research). The instrument is equipped with a He-Ne laser (633 nm) probing the surface at an incident angle of 70°. A single layer calculation model was used to quantify the layer thickness of TA coatings. Therefore, the refractive index of tannic acid coatings was assumed to be equal to SiO_2 (n_R = 1.468). For each time point, three measurements on three individual wafers were acquired and averaged (n_{rep} = 9). Si wafers were coated in groups (G_1: 0.5 h – 4 h; G_2: 8 h – 16 h; G_3: 24 h). The thickness of the native oxide layer was measured on control wafers and subtracted from the reported results.

AFM

An atomic force microscope (MFP 3D, Asylum Research) was used to determine the coating thickness by scratching the coatings with a cantilever (ACS-240TS) and scanning across the edge of the scratched area. Three measurements were conducted at random positions on the sample surface to yield an average value (n_{rep} = 3).

UV-vis

Quantification of the oxidation of TA solutions was performed using a Lambda 25 spectrophotometer (Perkin Elmer). Spectra were recorded with a resolution of 1 nm. All samples were filtered through a 0.2 μm polyether sulfone syringe filter prior to the measurement. Due to strong adsorption in the UV-region samples were diluted 1/100 in their corresponding buffer solutions.

Supporting information for solid-state NMR measurements

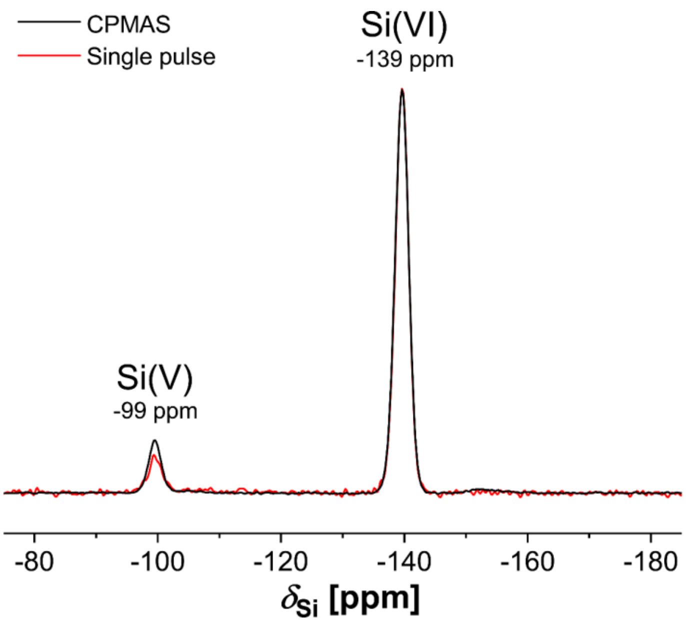

Figure S1: ^{29}Si NMR spectra recorded at B_0 = 9.4 T from the TA_{prec} sample, using either direct excitation by single pulses at 14.00 kHz MAS (red trace) or $^{1}H\rightarrow^{29}Si$ CP at 7.00 kHz (black trace). Note (*i*) the almost identical NMR-peak intensites/widths observed between the quantitative single-pulse NMR spectrum and that recorded using CP with a contact period of 4.0 ms, as well as (*ii*) the very similar CPMAS NMR spectra revealed for the TA_{prec} and $TA_{coating}$ samples in Figure 1. Altogether, this strongly suggests essentially identical relative Si(V) and Si(VI) populations in TA_{prec} and the TA-coated TiO_2 nanoparticles: these results justify using the NMR results from the TA_{prec} specimen as representative also for the $TA_{coating}$ sample, whose minute Si content (see Table S2) precludes any NMR experimentation other than the $^{1}H\rightarrow^{29}Si$ CP NMR results shown in Figure 1. Note that no traces of silica (which produces ^{29}Si resonances around –110 ppm[7]) is revealed from any of the NMR spectra shown above and in Figure 1.

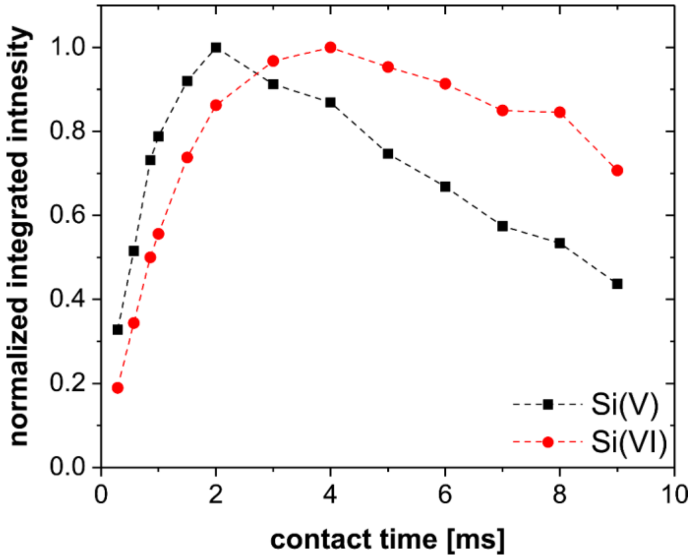

Figure S2: Integrated ^{29}Si CPMAS NMR peak-intensities plotted against the CP contact time-period for the resonances at –99 ppm and –139 ppm, which are associated with the ^{29}Si(V) and ^{29}Si(VI) sites of the TA_{prec} sample, respectively; see Figure S1. Each curve is normalized to a maximum integrated intensity of unity. The initial buildup-rate of the ^{29}Si NMR signal of each ^{29}Si(V) and ^{29}Si(VI) site grows as the inverse cube of its respective (*shortest*) $^{1}H–^{29}Si$ internuclear distance. Note the more rapid NMR-signal buildup from the ^{29}Si(V) sites relative to their ^{29}Si(VI) counterparts, as is expected from the presence of a Si(V)–OH linkage (as opposed to Si(VI)–O–C motifs only) in Scheme 1. We refer to Kolodziejski and Klinowski for general information about CPMAS NMR experimentation and its kinetics.[8]

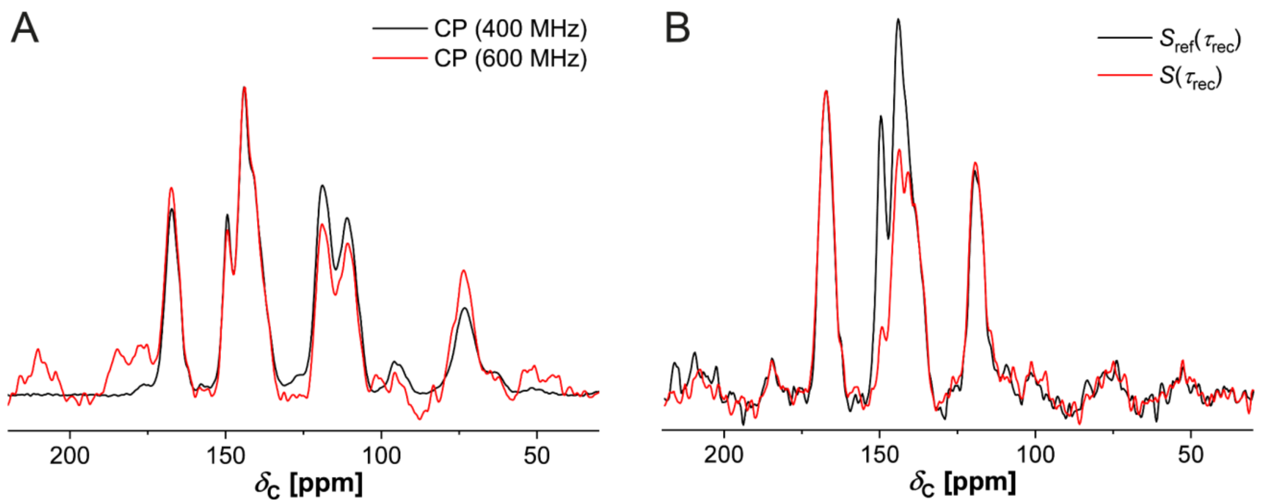

Figure S3: (A) ^{13}C CPMAS NMR spectra recorded from the TA$_{prec}$ sample, and obtained either at $B_0 = 9.4$ T and $\nu_r = 14.00$ kHz, or at $B_0 = 14.1$ T and $\nu_r = 10.00$ kHz. (B) ^{13}C{^{29}Si} REDOR NMR spectra acquired at $B_0 = 14.1$ T and $\nu_r = 10.00$ kHz, using a dipolar recoupling period of $\tau_{rec} = 4.0$ ms, during which ^{13}C–^{29}Si dipolar interactions are activated: they lead to an NMR-signal attenuation ("dephasing") from all ^{13}C sites in close proximity to ^{29}Si [$S(\tau_{rec})$; red trace] relative to the "reference" portion of the REDOR protocol [$S_{ref}(\tau_{rec})$; black trace]. A significant dephasing is only observed for the ^{13}C resonances in the 135–50 ppm spectral range; they are associated with aromatic ^{13}C–O moieties of TA,[9] thereby evidencing their closer ^{13}C–^{29}Si distances relative to those of all other ^{13}C sites. The signal ~150 ppm reveals the strongest attenuation, and is therefore attributed to ^{13}C–O–Si motifs (also see Figure S4). The absence of ^{13}C resonances in the 50–110 ppm range in the REDOR NMR spectra [compare with the ^{13}C CPMAS spectrum in (A)] is attributed to a rapid T_2 relaxation of these ^{13}C sites. Consequently, our data do not permit drawing any conclusion about their proximities to Si.

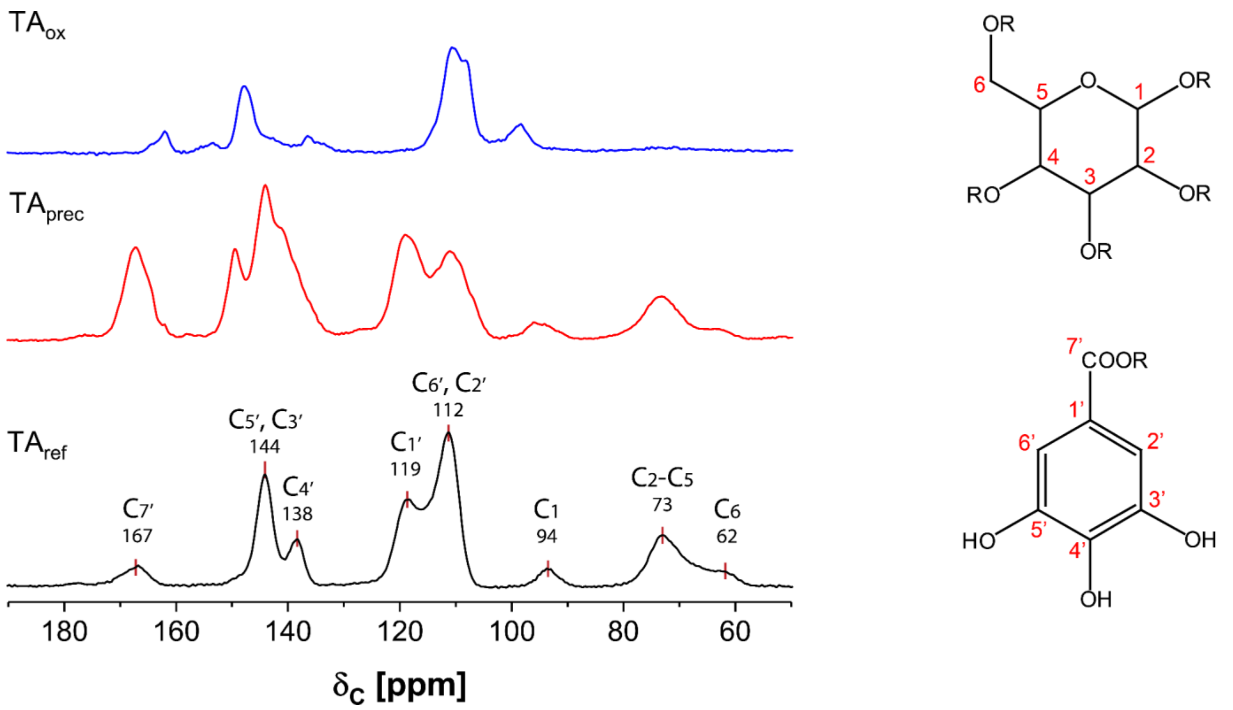

Figure S4: ^{13}C CPMAS NMR spectra recorded at $B_0 = 9.4$ T and 14.00 kHz MAS from pristine tannic acid (TA$_{ref}$), TA precipitated in presence of 1000 μM Si$_{aq}$ (TA$_{prec}$), as well as the oxidative polymerization product (TA$_{ox}$). The indicated ^{13}C chemical shifts (in ppm) and peak assignments in the NMR spectrum of TA$_{ref}$ refer to the C sites of the TA molecules labeled in the right panel.[9] Note the overall similar NMR results observed from the TA$_{ref}$ and TA$_{prec}$ samples, which mainly differ in the signal at δ_C ~150 ppm. This resonance is only observed from TA$_{prec}$ and is attributed to ^{13}C–O–Si moieties (see Figure S3), as further supported by ^{13}C chemical shifts reported from solution NMR by Evans et al.[10]

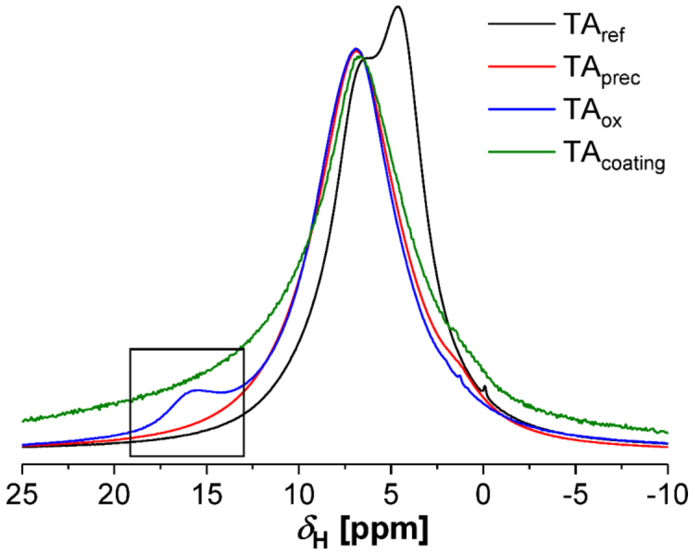

Figure S5: ^{1}H NMR spectra recorded at B_0 = 9.4 T and 14.00 kHz MAS from samples of pristine tannic acid (TA$_{ref}$), tannic acid precipitated in presence of 1000 μM Si$_{aq}$ (TA$_{prec}$), oxidative polymerization products of tannic acid (TA$_{ox}$), and TA coated TiO_2 particles prepared in HEPES buffer (pH = 7.8) together with Si at a concentration of 80 μM (TA$_{coating}$). Note the minor peak at 15.5 ppm in the NMR spectrum from TA$_{ox}$, which is attributed to hydrogen-bonded carboxy moieties.

Supporting information for surface analysis

XPS

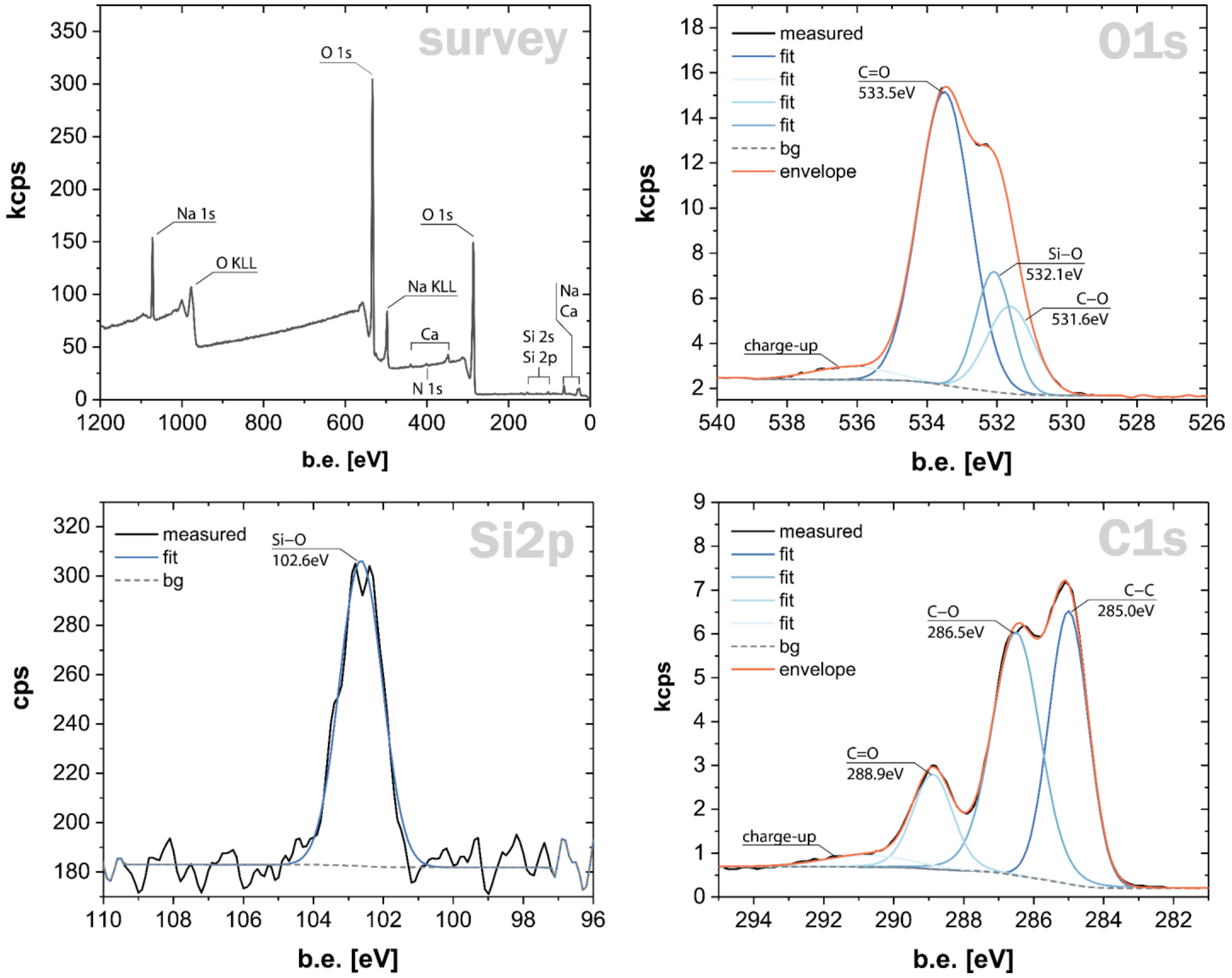

Figure S6: Chemical composition of TA coatings on titanium coins obtained in Bicine buffer supplemented with 80 μM Si_{aq} at pH = 7.8 determined by XPS. The survey scan indicated Na in the organic film, which may act as counter ions of deprotonated hydroxyl groups. Further, some Ca impurities are present. The high-resolution spectra of O 1s indicated three oxygen species, which were attributed to C=O, C–O, and Si–O bonds. The Si 2p peak is centered at 102.6 eV. Whilst the chemical state of Si could not be determined with sufficient reliability, the binding energy of Si excludes a pure SiO_2 phase.[11] It may rather be correlated to the bond length of hexa-coordinated Si–O–C bonds (Table S2). The C 1s spectrum shows three peaks indicating C=O, C–O, and C–C bonds.

Table S2: Correlation of bond length and binding energy in silicon compounds.

Type	Si–O (quartz)	Si–C	Si–O–R (hexa-coord.)	Si–Si
Bond length [pm]	161[12]	187.5[13]	170–190[14]	233[13]
Binding energy Si 2p [eV]	103.9[15]	102.5[15]	102.6[a]	99.3[16]

[a]*determined for Si in TA coatings on titanium surfaces*

Si distribution determined by XPS, ToF-SIMS, and EDS

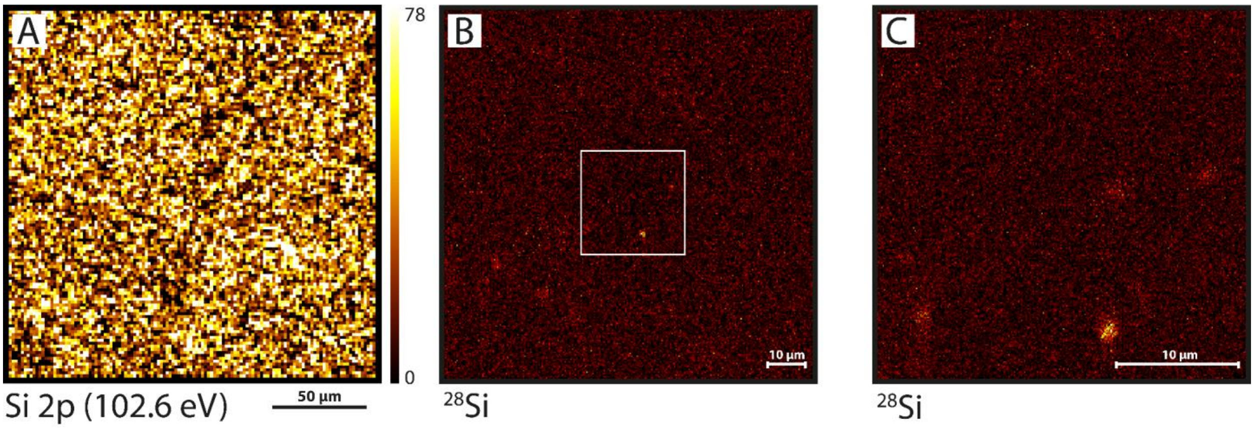

Figure S7: Representative distribution of Si in TA coatings on titanium coins from microscopic to submicroscopic scale: (A) Mapping of the Si 2p peak (102.6 eV) by XPS on an area of 200 × 200 µm. (B) ToF-SIMS mapping of ^{28}Si on an area of 100 × 100 µm and (C) subsequent high-resolution imaging of the center region. All measurements were performed on titanium coins coated with TA. The coating was obtained in Bicine buffered solution at pH = 7.8 supplemented with 80 µM Si_{aq} and a coating time of 24 h. The spacial resolution of 3 µm in XPS measurements did not allow to detect SiO_2 particles. However, the surface sensitive technique demonstrates the distribution of Si species throughout the measured area. This result was confirmed by high-resolution ToF-SIMS. Additionally, the higher spacial resolution detected some Si-rich areas which originate in particles as observed in SEM/EDS measurements (Figure S8), were detected.

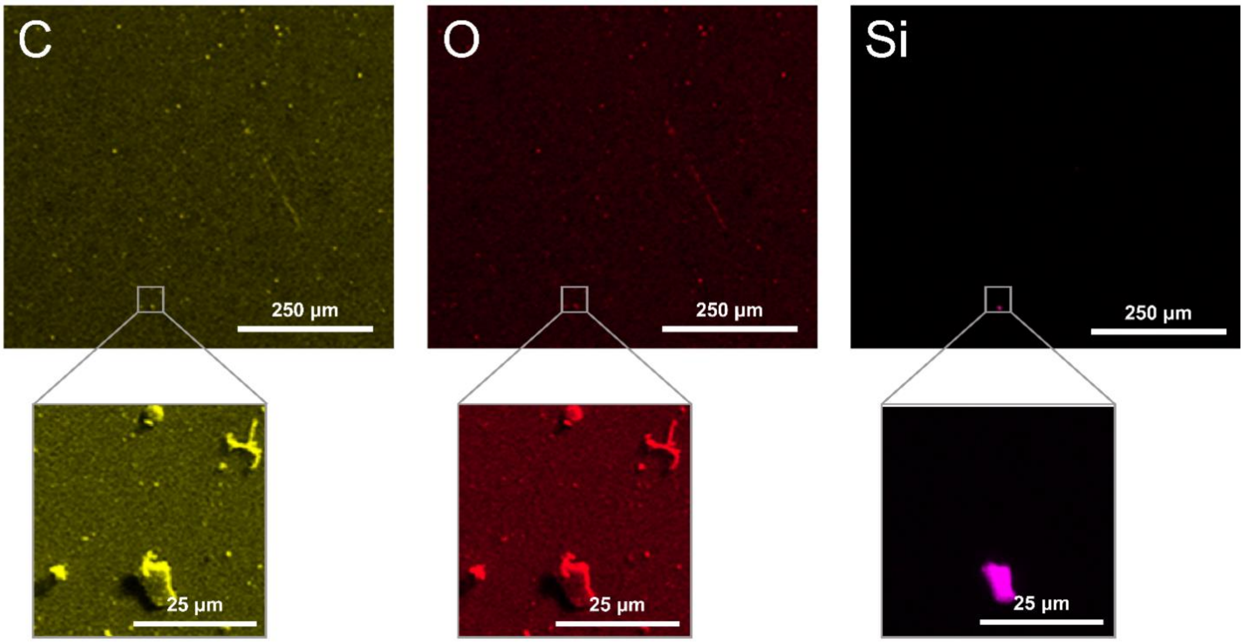

Figure S8: Representative EDS map of a Si-rich particle found on titanium surfaces coated with TA. The coating was obtained in Bicine buffer at pH = 7.8 for 24 h. A weak Si background signal was still obtained throughout the surface but the presence of particles suppressed the intensity in the false color map. The particles might originate from glassware or from the Si stock solution as ortho-silicic acid polymerization product.

Table S3: EDS-derived elemental contents (wt. %) of the samples used for solid-state NMR experiments.

Sample	[Si][a]	C	O	Si	Ti
TA_{prec}	1000 µM	48.8	46.2	2.3	–
$TA_{coating}$	80 µM	3.2	38.2	0.1	58.1

[a]*Nominal Si concentration during preparation.*

Supporting information for deposition of tannic acid coatings

QCM-D raw data

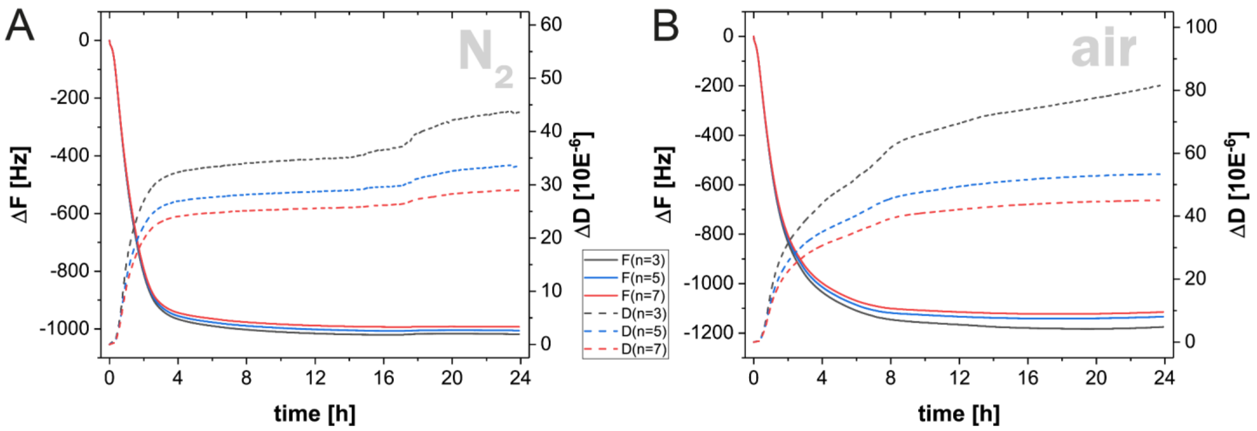

Figure S9: Averaged (n_{rep} = 3) progression of frequency and dissipation shifts of the 3rd, 5th, and 7th overtone (*n*) in QCM-D during deposition of TA on titanium sensors. Adsorption of TA from Bicine buffer at pH = 7.8 under depletion of oxygen (A) in comparison to the oxidative environment (B). Bicine buffer at pH = 7.8 was degassed prior to the experiment and constantly bubbled with nitrogen throughout the measurement. Under this condition, the colorless solution indicated no oxidation of TA. The reduced particle formation by oxidative polymerization also led to a more stable dissipation value in QCM-D during the course of a 24 h deposition process (note the different scale of the y-axes). The result indicates that oxidative polymerization by dissolved O_2 is not required for the formation of silicate-TA networks.

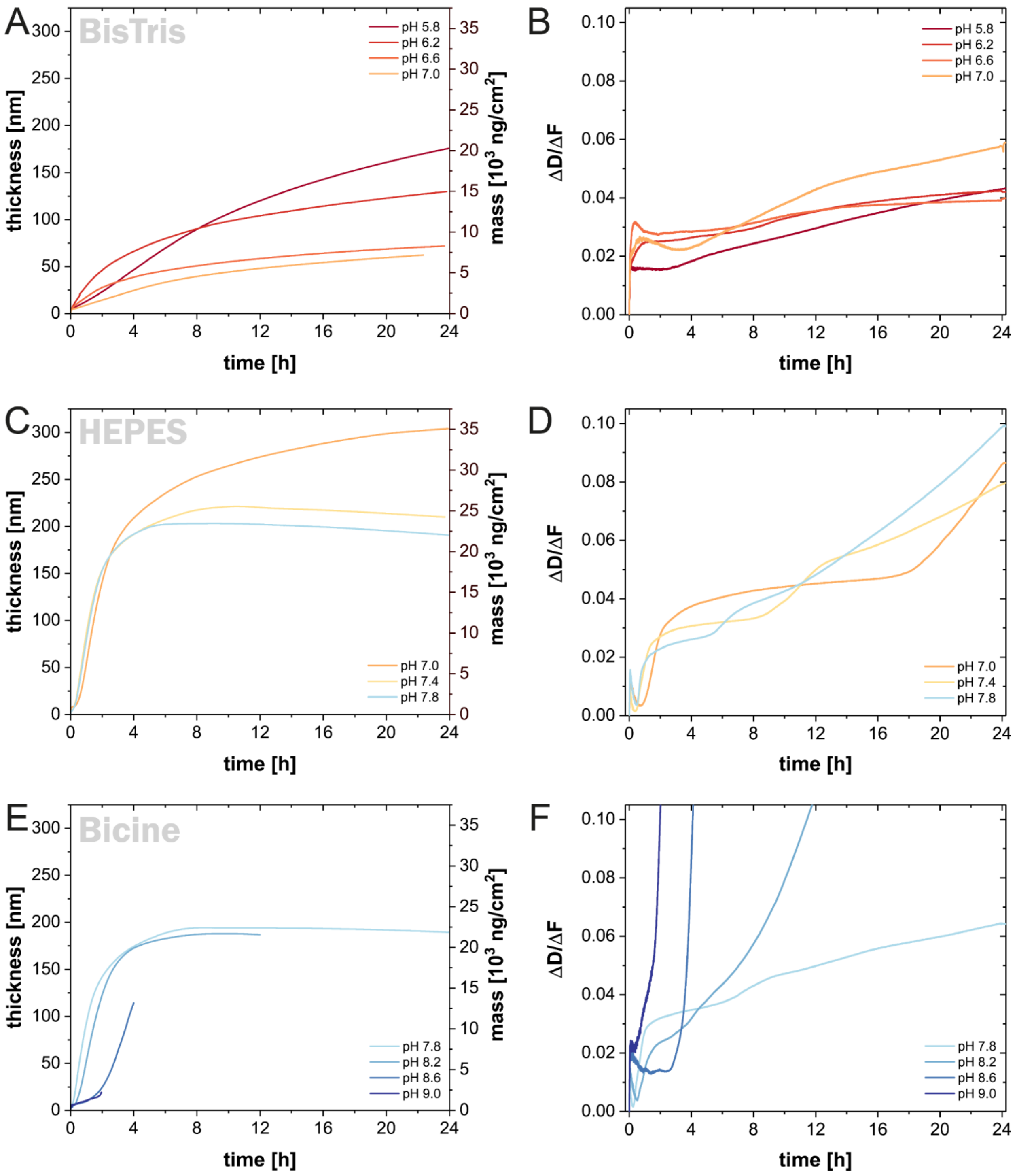

Figure S10: Averaged (n_{rep} = 3) progression of TA coating thickness calculated with the Voigt viscoelastic model (A, C, E). Additionally, the ratio of $\Delta D/\Delta F$ [10^{-6}Hz^{-1}] of the 3rd harmonic overtone is given in the panels to the right (B, D, F). Due to rapid oxidation and polymerization processes at pH above 8.6, the deposition efficiency was greatly impeded and the process was aborted after 2 h (pH = 8.6) and 1 h (pH = 9.0). Splitting overtones and increasing $\Delta D/\Delta F$ values were associated with the formation of polymeric byproducts. These polymers agglomerate and form particles, which sediment onto the sensor surface.[17]

Spectrophotometric determination of TA oxidation

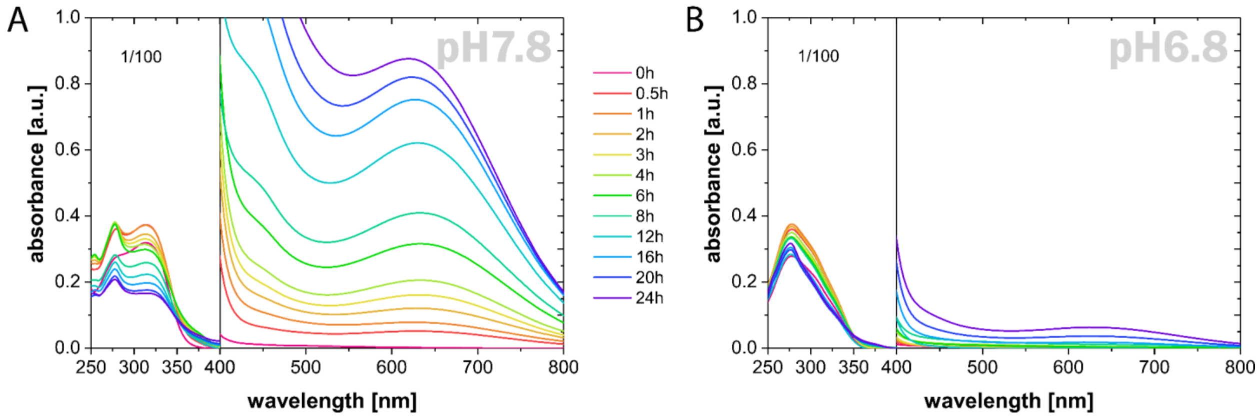

Figure S11: Representative progress of TA oxidation measured by UV-vis spectrophotometry. TA was dissolved in HEPES buffer at pH = 7.8 (A) and 6.8 (B). Solutions were exposed to air and gently stirred (100 rpm) during the course of 24 h. Prior to analysis, solutions were filtered through a 0.2 μm syringe filter. Due to strong adsorption in the UV region, samples were diluted (1/100) with buffer. The increased adsorption in the visible range is associated with the oxidation of the polyphenolic molecules.[18]

NPS data

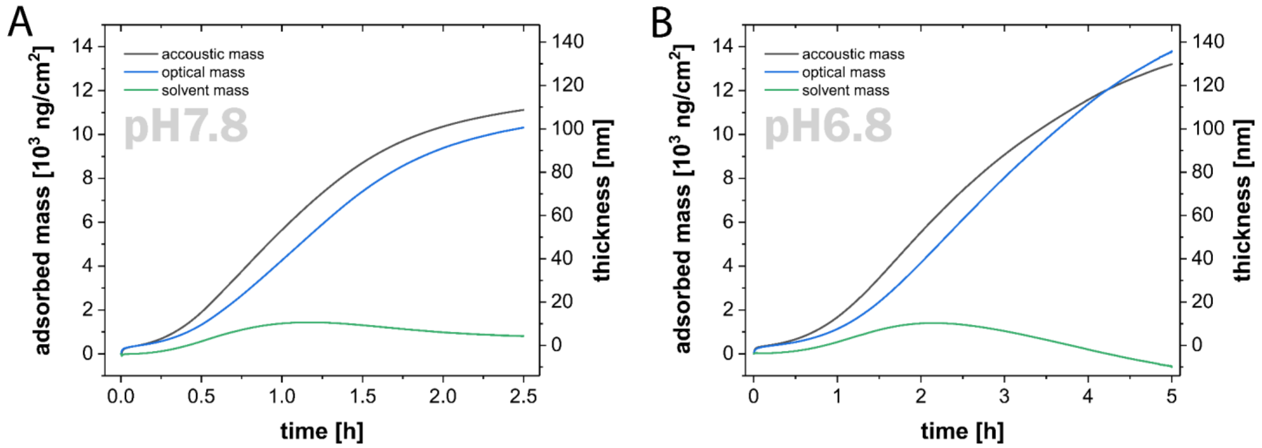

Figure S12: Representation of optical mass and acoustic mass of TA coatings determined by NPS and QCM-D respectively (n_{rep} = 2). Both conditions pH = 7.8 (A) and pH = 6.8 (B) show low hydration during initial formation of the coating followed by an increase. Once the penetration depth of NPS exceeds its characteristic probing depth of 30 nm the correlation starts to deviate and the estimation of the hydration decreases. Above 100 nm Equation S1 is no longer valid and the data cannot be interpreted correctly.

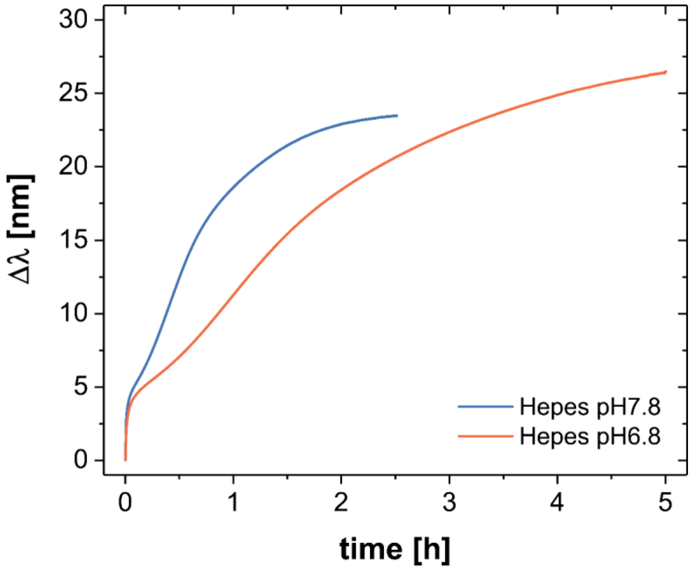

Figure S13: Averaged (n_{rep} = 2) shift of plasmon peak during the deposition of TA at pH = 6.8 and pH = 7.8. The measurements were conducted until the peak shift leveled off, indicating that the maximum penetration depth of the surface plasmon was reached. The variation in maximum peak shift might indicate a differing refractive index of layers obtained in oxidizing condition (pH = 7.8) compared to non-oxidizing conditions (pH = 6.8).

CSTR type deposition of TA

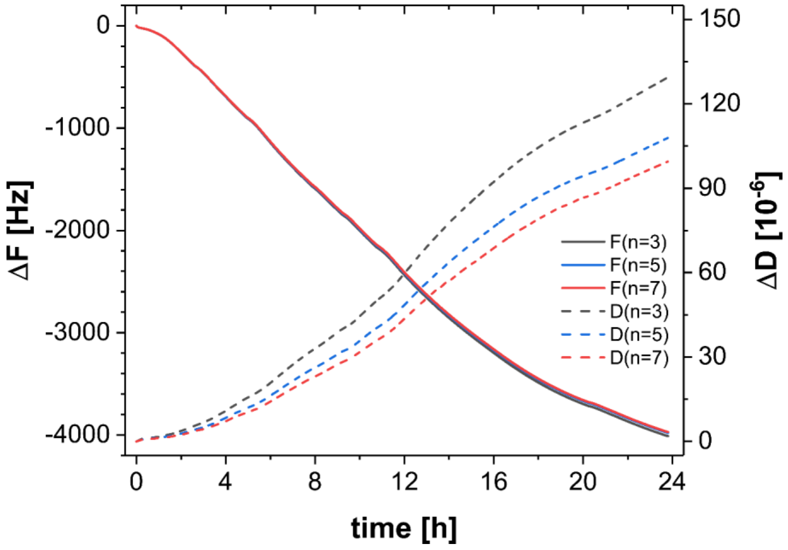

Figure S14: Averaged (n_{rep} = 4) and normalized frequency and dissipation shifts of the 3rd, 5th, and 7th QCM-D overtone (*n*) as a function of time. Graphs show the deposition of TA onto Ti surfaces in a continously stirred tank reactor (CSTR) setup with TA dissolved at pH = 6.8 and fed with Si_{aq} in alkaline condition (pH = 8.8) to result in a final pH-value of 7.8. The continuous deposition could be maintained throughout 24 h, owing to the reduced particle formation in the CSTR. Slight interferences in the slope of the QCM-D measurement were due to a change in reactant volume in the CSTR. The operation with a single peristaltic pump caused invariances in in- and out-flow of the CSTR (Figure S16).

TA deposition from inorganic buffers

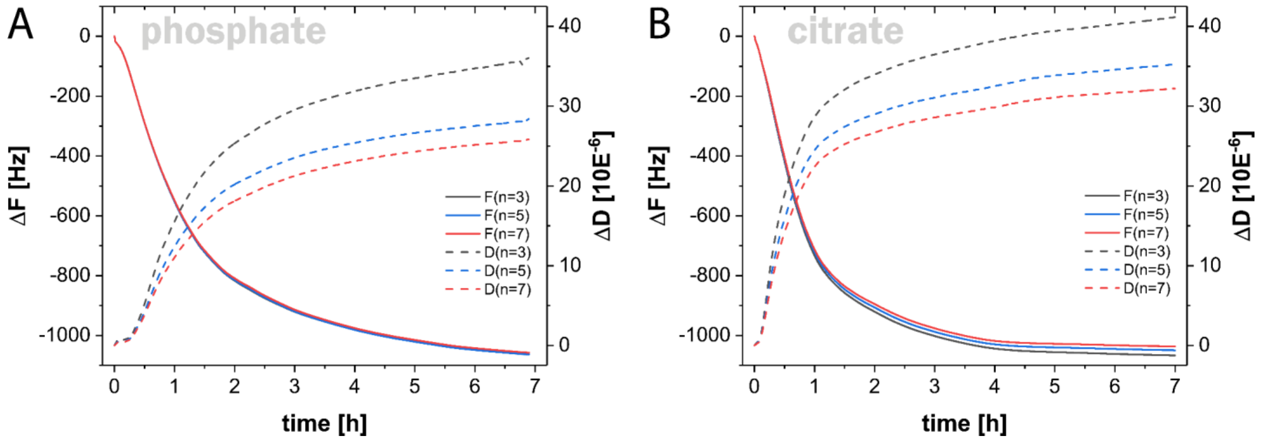

Figure S15: Averaged (n_{rep} = 3) progression of frequency and dissipation shifts of the 3rd, 5th, and 7th overtone (*n*) in QCM-D during deposition of TA on titanium sensors. Adsorption of TA from phosphate buffer (A) and citrate/phosphate buffer (B) that were both supplemented with 600 mM NaCl and 80 μM Si_{aq} at pH = 7.0.

CSTR setup

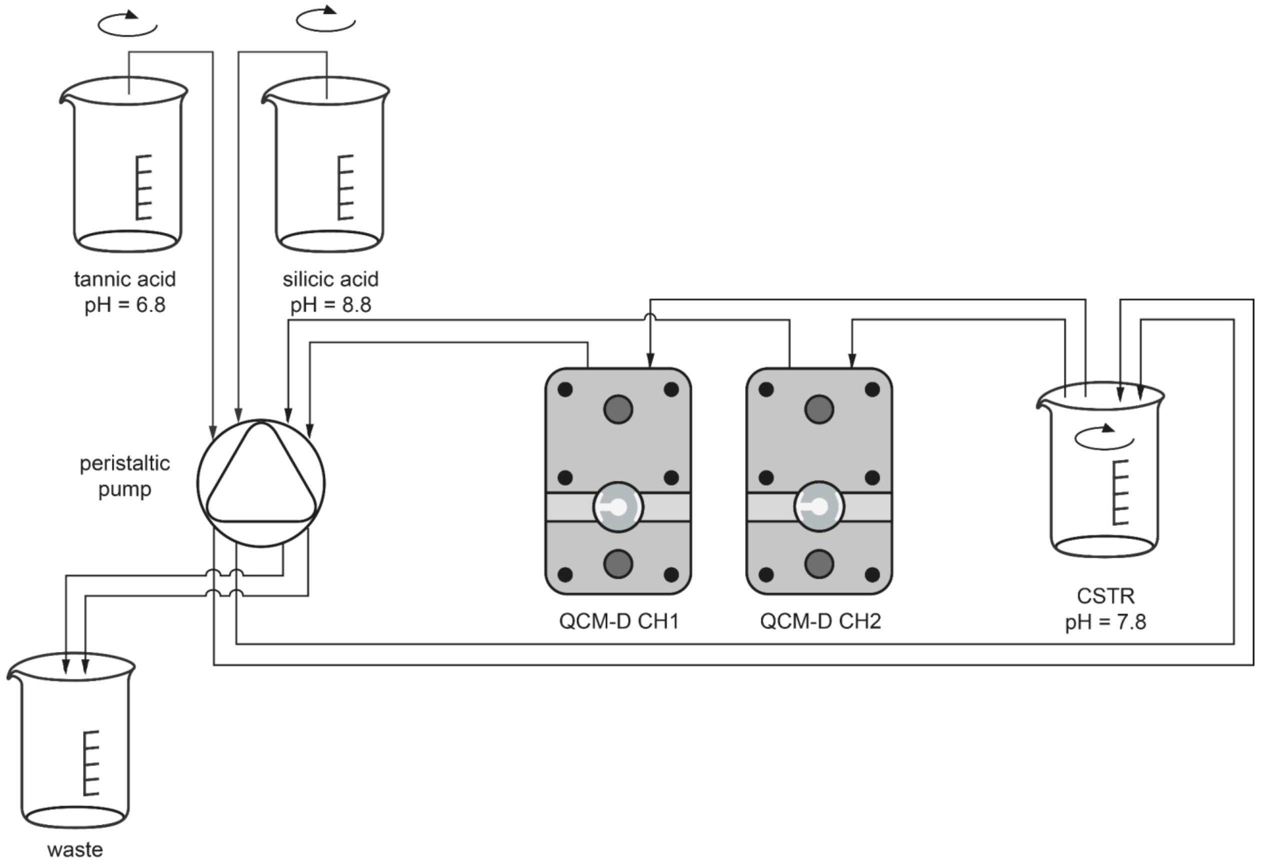

Figure S16: Flow chart of the continously stirred tank reactor (CSTR) setup for QCM-D measurements shown in Figure S14. Tannic acid was kept at mild acidic pH = 6.8 separated from silicic acid in alkaline condition. Mixing in the CSTR at a flow rate of 0.1 ml/min and a reaction volume of 6 ml resulted in an average residence time of 30 min. The solution in the CSTR was set to pH = 7.8 by adjusting the pH level of the silicic acid feed. The concentrations of TA and Si_{aq} in the CSTR were set equally to other QCM-D experiments to 1 mg/ml and 80 μM respectivly.

References

[1] G. Metz, X. L. Wu, S. O. Smith, *J. Magn. Reson. Ser. A* **1994**, *110*, 219.
[2] B. M. Fung, A. K. Khitrin, K. Ermolaev, *J. Mag. Reson.* **2000**, *142*, 97.
[3] T. Gullion, J. Schaefer, *J. Mag. Reson.* **1989**, *81*, 196.
[4] T. Gullion, D. B. Baker, M. S. Conradi, *J. Mag. Reson.* **1990**, *89*, 479.
[5] F. Weber, A. Barrantes, H. Tiainen, *Langmuir* **2019**, *35*, 3327.
[6] M. P. Jonsson, P. Jönsson, F. Höök, *Anal. Chem.* **2008**, *80*, 7988.
[7] M. Edén, *Annu. Rep. Prog. Chem., Sect. C: Phys. Chem* **2012**, *108*, 177.
[8] W. Kolodziejski, J. Klinowski, *Chem. Rev.* **2002**, *102*, 613.
[9] a) T. Kraus, Y. Zc, C. M Preston, R. A. Dahlgren, R. J Zasoski, *J. Chem. Ecol.* **2003**, *29*, 703; b) F. H. Romer, A. P. Underwood, N. D. Senekal, S. L. Bonnet, M. J. Duer, D. G. Reid, J. H. van der Westhuizen, *Molecules* **2011**, *16*, 1240.
[10] D. F. Evans, A. M. Z. Slawin, D. J. Williams, C. Y. Wong, J. D. Woollins, *J. Chem. Soc., Dalton Trans* **1992**, 2383.
[11] D. S. Jensen, S. S. Kanyal, N. Madaan, M. A. Vail, A. E. Dadson, M. H. Engelhard, M. R. Linford, *Surf. Sci. Spectra* **2013**, *20*, 36.
[12] A. F. Wells, *Structural inorganic chemistry*, 5th ed ed., Clarendon Press; New York: Oxford University Press, Oxford, **1984**.
[13] B. Beagley, J. J. Monaghan, T. G. Hewitt, *J. Mol. Struct* **1971**, *8*, 401.
[14] a) R. Tacke, A. Stewart, J. Becht, C. Burschka, I. Richter, *Can. J. Chem.* **2000**, *78*, 1380; b) K. Junold, J. A. Baus, C. Burschka, T. Vent-Schmidt, S. Riedel, R. Tacke, *Inorg. Chem.* **2013**, *52*, 11593; c) P. Bindu, B. Varghese, M. N. S. Rao, *Phosphorus Sulfur Silicon Relat. Elem.* **2003**, *178*, 2373.
[15] K. M. R. Kallury, U. J. Krull, M. Thompson, *Anal. Chem.* **1988**, *60*, 169.
[16] National Institute of Standards and Technology, https://www.nist.gov/, *(accessed 07 Jan 2019).*
[17] D. Johannsmann, I. Reviakine, R. P. Richter, *Anal. Chem.* **2009**, *81*, 8167.
[18] E. Haslam, in *Practical Polyphenolics: From Structure to Molecular Recognition and Physiological Action*, Cambridge University Press, **1998**.
